# Resilience in People with Lewy Body Disorders and Their Care Partners: Association with Mental Health, Relationship Satisfaction, and Care Burden

**DOI:** 10.3390/brainsci12020148

**Published:** 2022-01-23

**Authors:** Sabina Vatter, Iracema Leroi

**Affiliations:** 1 School of Psychology, Faculty of Science, The University of Sydney, Sydney, NSW 2050, Australia; sabinavatter@gmail.com; 2 Global Brain Health Institute, Trinity College Dublin, D02 PN40 Dublin, Ireland

**Keywords:** resilience, Parkinson’s disease dementia (PDD), Lewy body dementia (LBD), mild cognitive impairment in Parkinson’s (PD-MCI), informal care, Lewy body diseases, cognitive impairment, care burden, anxiety, depression

## Abstract

The emergence of cognitive impairment and dementia in people with Lewy body spectrum disorders (LBS) significantly impacts the quality of life of the individual and their care partner. Coping well with the condition may depend, in part, on the degree of psychological resilience or capacity to ‘bounce back’ from adversity. We explored resilience in people with Parkinson’s disease mild cognitive disorder or dementia, or dementia with Lewy bodies, and their care partners, and its relation to outcomes related to their mental well-being and quality of life. This exploratory, cross-sectional study recruited 76 participant-dyads. Resilience, quality of life, depression, anxiety, and relationship satisfaction were evaluated in both members of the dyad. In care partners, care burden and stress were also assessed. Over 70% of both care partners and recipients reported high levels of resilience. Lower resilience in both members of the dyad was associated with higher anxiety and lower quality of life. Additionally, lower resilience in care partners was associated with lower well-being, relationship satisfaction, and higher burden and stress. Resilience in persons with LBS and their care partners is important to consider when assessing mental health, relationship, and care burden outcomes, acting as a focus of intervention to support positive outcomes.

## 1. Introduction

The spectrum of Lewy body diseases comprises disorders characterised by cognitive impairment, ranging from mild cognitive impairment in Parkinson’s disease (PD-MCI) to dementia (Parkinson’s disease dementia (PDD); dementia with Lewy bodies (DLB)). Collectively, PDD and DLB constitute over 15% of total dementias [1], and DLB is considered the second most common type of neurodegenerative dementia following Alzheimer’s disease [2]. As these conditions advance, and the stage of cognitive impairment progresses, the negative impact of the condition on those affected, as well as their care partners, becomes significant, often manifesting as lower quality of life in both members of the dyad, higher levels of disability, and increasing care burden [3,4,5]. Thus, the need for moderating factors to optimise quality of life and well-being is important. One of these protective factors may be psychological resilience, broadly defined as the capacity to bounce back from adversity [6], maintain psychological health, and adapt and grow in the context of adversity [7,8]. It has been suggested that the presence of resilience enables care partners to continue to provide care and even flourish despite the increasing demands of a progressive condition in the care recipient [9].

Investigations of resilience have generally focused on children and younger populations facing adverse circumstances (i.e., [6]). In older adults, resilience work has mostly centred on the response to physical illness [10,11,12,13] and traumatic events [14]. In Parkinson’s disease, there have been only a few studies [15,16,17,18,19,20], and in dementia, the focus has been on care partners [21] rather than people with dementia themselves [22,23]. To date, no studies have addressed Lewy body spectrum disorders with cognitive impairment. Thus, here, we focus on both the person with Lewy body-related cognitive disorders (PD-MCI, PDD, or DLB), as well as their care partner, considering resilience in the context of the care dyad, considering the reciprocal impacts mental states have on each partner of the dyad [24]. Resilience research supports the positive psychology approach [25], which, considering the progressive and incurable nature of the Lewy body spectrum disorders, can offer an alternative to a focus on the ‘broken brain’ and concepts of care burden and stress.

Resilience has been conceptualised as a multidimensional construct, including as a process or adaptation [26,27,28], an antecedent factor (i.e., risk or protective factor; [29]), a manifestation of neurobiological processes underpinning reward and motivation [30], and as an outcome or consequence. In a recent systematic review exploring resilience in family caregivers of people living with chronic neurological conditions, the authors concluded that due to various studies referring to resilience as a process, trait, or a hybrid of the two, it is next to impossible to achieve consensus on how resilience is conceptualised, theorised, and evaluated [31]. It comprises factors such as mental well-being (i.e., absence of depression and anxiety), self-efficacy, hope, self-confidence, ability to problem solve, coping ability, degree of social support, and optimism [6,8,26,32]. Its multidimensionality makes measuring resilience challenging; however, validated tools to measure resilience, such as the Brief Resilience Scale (BRS) [33], can still provide valuable insights.

To investigate resilience in the context of the Lewy body spectrum disorders, we undertook a detailed exploratory investigation in people with PD-MCI, PDD, and LDB, as well as their care partners, focussing on the interplay among resilience, depression, anxiety, quality of life, relationship satisfaction, and care burden. For the purposes of this study, we conceptualised resilience as a predictor or antecedent of other measures, including mental health, relationship satisfaction, and quality of life outcomes in both members of the dyad, and burden, stress, and strain in care partners. We applied the adapted Stress-Appraisal Model [34] as a theoretical framework for the hypotheses in this study. Specifically, the neuropsychiatric and cognitive symptoms of the person with PD act as primary stressors for the care partner, whereas their own mental and physical health as secondary stressors, which influence how they respond to the situation (primary and secondary appraisals). The outcome may in part depend on the protective and mediating factors, such as quality of dyadic relationship, perceived social support, self-efficacy, and personality (including resilience), leading to the tertiary appraisal (e.g., burden) and outcome (e.g., quality of life). We hypothesised that lower resilience predicts lower mental well-being, quality of life, and relationship satisfaction in both members of the care dyad. Moreover, we hypothesised that in care partners, lower resilience predicts higher stress and burden. Our overall aim was to gather data to inform the development of dyadic psychosocial interventions for people with Lewy body-related cognitive disorders and their care partners.

## 2. Materials and Methods

### 2.1. Context, Design, and Ethics

This was a cross-sectional study nested in the INdiVidualised cognitivE Stimulation Therapy (INVEST) project, a pilot feasibility randomised controlled trial of an adapted cognitive stimulation therapy for people with Lewy body-related cognitive disorders and their study partners [35,36,37].

The study was approved by the Yorkshire and Humber-Bradford Leeds Research Ethics Committee (Reference: 15/YH/0531) and was conducted according to standards set by the Declaration of Helsinki and the principles of Good Clinical Practice.

### 2.2. Participants

We included 76 participant-dyads who were people in different stages of cognitive impairment due to PD-MCI, PDD, or DLB (*n* = 76), and their care partners (*n* = 76). Participants with Lewy body-related cognitive disorders were eligible if they were: aged 18 years or older; having a diagnosis based on standard clinical diagnostic criteria of PD-MCI [38], PDD [39], or DLB [40] ascertained by the referring specialist; living at home; on stable medication for at least four weeks prior to study entry; had the capacity to provide consent to participate in the study; and had the ability to speak and understand conversational English [35]. Exclusion criteria were the presence of a medical, psychiatric, or cognitive illness severe enough to interfere with study procedures and lack of care partner willing to participate. Inclusion criteria for care partners were at least 18 years old, being the primary person responsible for unpaid support/care for the participant with Lewy body-related cognitive disorders at least four hours per week in the community, willing to be a coparticipant in the study, and able to speak and understand English. All participants were assessed for capacity to consent to the study and signed informed consent. The participant-dyads were identified for the INVEST study via seven health and social care organisations across England. All study visits were conducted in participants’ own home by researchers trained in standard administration of the outcome measures and scoring procedures.

### 2.3. Measures

All participant-dyads completed a battery of measures (detailed description in Table 1). Demographic information (e.g., age, gender, education, ethnicity, marital status, relationship duration, living status of both partners), disease-related aspects of the person with Lewy body-related cognitive disorders (e.g., diagnosis, onset year of PD or DLB symptoms and cognitive impairment), and care provision-related aspects of the care partner (e.g., care provision duration in years and weekly hours) was obtained from all participants. Both members of the dyad were administered the Brief Resilience Scale (BRS) [33], which is a 6-item scale that assesses the ability to bounce back or recover from stress on a five-point Likert scale (from 1—strongly disagree to 5—strongly agree), with higher scores indicating higher resilience. Three items are worded positively (for example: “I usually come through difficult times with little trouble”), and three items are worded negatively (for example: “I have a hard time making it through stressful events”). In care partners of people with Lewy body-related cognitive disorders, the BRS demonstrates strong psychometric and clinimetric properties and excellent clinical utility ratings [41].

To capture the dyadic aspect of the care relationship, we administered the Relationship Satisfaction Scale (RSS) [42], which is a seven-item scale rated on a Likert scale (ranging from 0—very dissatisfied to 6—very satisfied) exploring communication and openness, resolving conflicts and arguments, degree of affection and caring, and overall satisfaction with the relationship. It has been used in this population before [4,35] and has also demonstrated good psychometric and clinimetric properties [41]. All participants also completed the Hospital Anxiety and Depression Scale (HADS) [43]. In addition, care partners completed the Neuropsychiatric Inventory (NPI) [44] rating the psychiatric symptoms of the person with Lewy body-related cognitive disorder.

To explore the relationship between resilience and quality of life, we administered the Parkinson’s Disease Questionnaire-39 (PDQ-39) [45] in participants with Lewy body-related cognitive disorders, and all dyads completed the *EuroQoL-5D-3L* (EQ-5D) [46]. Additionally, to characterise clinical aspects of participants with Lewy body-related cognitive disorders, we administered the Montreal Cognitive Assessment (MoCA) [47], Hoehn and Yahr staging system (H&Y) [48], the Unified Parkinson’s Disease Rating Scale (UPDRS-III) [49], and the Schwab and England Activities of Daily Living (SE-ADL). Finally, care partners also completed the Zarit Burden Interview (ZBI) [50], the Relatives’ Stress Scale (Rel.SS) [51], and the Short-Form 12 Health Survey (SF-12) [52].

### 2.4. Analysis

Categorical variables (i.e., descriptive data) are presented as percentages, and normally or non-normally distributed continuous variables are presented as mean and standard deviations (SD) or as medians and interquartile ranges (IQRs), respectively. Parametric tests (i.e., regression analysis) and nonparametric tests (i.e., Spearman correlation coefficient, Mann–Whitney *U* test) were undertaken as appropriate. Assumptions for linear regression were examined via statistical tests and visual inspection of graphs and were met. Post hoc tests (i.e., Bonferroni) were applied when using several tests and several groups. Missing data were addressed with the expectation–maximisation method. The analyses were conducted in SPSS version 23. The significance level for all results was set at *p* < 0.05.

## 3. Results

### 3.1. Demographic and Clinical Characteristics

Participant-dyad characteristics are outlined in Table 2. Of the participants with Lewy body cognitive disorders, 19.8% (*n* = 15) had a diagnosis of PD-MCI, 52.6% (*n* = 40) had PDD, and 27.6% (*n* = 21) had DLB. Of this group, 78.9% (*n* = 60) were male, and 93.4% (*n* = 71) were white with a mean age of 74.5 years (SD = 6.74). Of the care partners, 85.6% (*n* = 65) lived with their study partner, 77.6% (*n* = 59) were spouses or partners, 17.1% (*n* = 13) were relatives, and the remainder 5.3% (*n* = 4) included a live-in care partner, a live-in divorcee, a friend, and a grandchild. Of the care partners, 89.5% (*n* = 68) were female, and 92.1% (*n* = 70) were white with a mean age of 65.0 years (SD = 11.81). Care partners provided care between 0 and 20 years (median = 3, IQR = 1–6.75), and half of the care partners (*n* = 39) provided up to 24 h of care every day (median = 71 h per week, IQR = 15.5–168).

### 3.2. Associations with and Predictors of Resilience

Table 1 describes the outcome measures and compares the group differences on various outcomes between people with Lewy body cognitive disorders and care partners. Group analysis (Mann–Whitney U test) revealed that care partners self-reported higher resilience scores (m = 3.79, SD = 0.82) than people with Lewy body cognitive disorders (m = 3.23, SD = 0.71, *p <* 0.001). People with Lewy body-related cognitive disorders reported lower quality of life (as measured by the EuroQoL) and higher anxiety and depression (as measured by the HADS) compared with care partners. Care partners were less satisfied with their relationship with the person with Lewy body-related cognitive disorders (m = 29.05, SD = 10.36) than the care recipients were with their care partner (m = 33.16, SD = 7.47, *p* = 0.016).

Table 3 shows the outcome scores of both members of the dyad according to the low (≤2.99) and high (≥3.00) resilience cut-off scores. Most participants with Lewy body-related cognitive disorders (74%; *n* = 56) and care partners (83%; *n* = 63) reported high resilience (i.e., above a mean score of 3.00). People with Lewy body-related cognitive disorders with lower levels of resilience had higher levels of anxiety (HADS, *p* < 0.001), higher frequency and severity of neuropsychiatric symptoms (NPI, *p* = 0.047), lower levels of quality of life related to Parkinson’s (PDQ-39, *p* = 0.006), and overall quality of life (EQ-5D, *p* = 0.004) compared to those with higher resilience scores. Care partners with lower levels of resilience reported lower relationship satisfaction (RSS, *p* = 0.002), lower quality of life (EQ-5D, *p* = 0.001), lower scores on mental health (SF-12-MCS, *p* < 0.001) and physical health (SF-12-PCS, *p* = 0.037), and higher levels of anxiety (HADS, *p* < 0.001), depression (HADS, *p* < 0.001), burden (ZBI, *p* < 0.001), and stress (Rel.SS, *p* < 0.001).

Table 4 and Table 5 outline the associations of resilience among people with Lewy body-related cognitive disorders and care partners, respectively, using Spearman rank correlation analyses (with Bonferroni-adjusted alpha levels of 0.003). Higher resilience in people with Lewy body-related cognitive disorders was associated with lower anxiety (HADS-A, *p* < 0.001) and higher overall quality of life (EQ5D-VAS, *p* < 0.001) and PD-related quality of life (PDQ-39, *p* = 0.001). In care partners, higher resilience was related to higher relationship satisfaction (RSS, *p* = 0.002), better mental health (SF-12-MCS, *p* < 0.001), and higher quality of life (EQ5D, *p* ≤ 0.002), as well as lower burden (ZBI), stress (Rel.SS), anxiety (HADS), and depression (HADS) (all at *p* < 0.001).

Table 6 reports the regression analysis. Individual linear regression models were built with regression being the predictor. In people with Lewy body-related cognitive disorders, resilience was the strongest predictor for anxiety level (F(1,74) = 19.97, *p* < 0.001, adjusted R2 = 0.20). Resilience was also a significant predictor for relationship satisfaction (F(1,74) = 4.21, *p* < 0.05, adjusted R2 = 0.04), quality of life (EQ5D-VAS: F(1,74) = 8.51, *p* < 0.01, adjusted R2 = 0.09), and Parkinson’s-related quality of life (PDQ-39: F(1,74) = 11.39, *p* < 0.01, adjusted R2 = 0.12).

In care partners, resilience was a strong predictor for several outcomes: anxiety (F(1,74) = 64.859, *p* < 0.001, adjusted R2 = 0.460), depression (F(1,74) = 31.849, *p* < 0.001, adjusted R2 = 0.291), overall mental health (SF12-MCS: F(1,74) = 31.009, *p* < 0.001, adjusted R2 = 0.286), stress (Rel.SS: F(1,74) = 27.290, *p* < 0.001, adjusted R2 = 0.260), and care burden (ZBI: F(1,74) = 24.749, *p* < 0.001, adjusted R2 = 0.240).

## 4. Discussion

As far as we are aware, this is the first investigation of resilience in people with Lewy body-related cognitive disorders and their care partners. We found, in support of our hypotheses, that higher resilience is associated with better mental health and higher quality of life in both members of the dyad and with lower care burden, stress, and strain in care partners. Relationship satisfaction, although reported as higher in care recipients than care partners, was associated with higher resilience in care partners. This underscores the importance of resilience as a potential protective factor in several outcomes in people with progressive neurodegenerative conditions such as Lewy body disorders, as well as their care partners. It provides evidence to support a shift in focus from care burden, stress, and strain towards positive and adaptive processes and may inform proactive and constructive approaches to support care dyads.

We found that care partners had higher resilience compared to care recipients. Adaptive changes in neural circuitry mediating mechanisms of reward, fear, and social behaviour play a role in resilience associated with an enhanced ability to cope with stress [30,53]. It is possible that the disruptions to reward and motivation pathways in people with Lewy body pathology, specifically Parkinson’s disease [54], may interfere with resilience networks, explaining the difference in resilience between our Lewy body disease group and their care partners, without an underlying neurodegenerative disorder. Work to identify the neurophysiological substrates that determine a predisposition to resilience to stress and depression is ongoing [55].

A systematic review exploring resilience in family care partners of people with dementia found that resilience is multidimensional and encompasses caring, social, cultural, and psychological dimensions of caring, all of which may influence care partners’ adaptability to their role of providing care [23]. The authors concluded that resilience may be care partners’ way of responding and adjusting to the new role of care partner as a condition progresses [23]. Indeed, as the health of people with Lewy body disorders progressively deteriorates, care partners have to accommodate and adapt to this journey, and assume the primary care partners’ role, evolving from their previous roles of spouse, life partner, child, sibling, or relative of the person with the degenerative condition. This adaptation requires mental and physical strength, as care partners’ time spent on caring tasks continually increases, and their own health and needs become deprioritised, increasing the risk of poor physical and mental health [4]. Our findings are in line with earlier studies, which have demonstrated that higher resilience is associated with lower care burden [56,57], potentially attributed to the positive benefits that care partners of people with Lewy body dementias experience by overcoming the many challenges of caring, despite high associated care burden [56]. Understanding resilience is therefore an important aspect influencing the extent of care burden experienced by care partners. Moreover, there are important implications for clinical practice. Applying a positive psychology lens and focussing on developing resilience in the therapeutic setting may foster better outcomes across a range of clinical symptoms.

Our study supported the association between high resilience and measures of better mental health, particularly anxiety, in both members of the dyad. It has been long hypothesised that resilience acts as a defence against adversity [58] and is thus protective of mental health. Participants with Lewy body-related cognitive disorders in the lower resilience group had higher frequency and severity of neuropsychiatric symptoms, although the direction of association cannot be inferred. As cognition in Parkinson’s deteriorates, neuropsychiatric symptoms emerge, with the frequency, magnitude, and range of neuropsychiatric symptoms, including apathy, being highest in the PDD stage [3,59]. It is possible that the presence of neuropsychiatric symptoms undermines resilience, or that lower resilience leads to the emergence of neuropsychiatric symptoms. This underscores the complexity of resilience studies in that it may be an antecedent factor (i.e., risk or protective factor; [29]), an outcome or consequence, or indeed a manifestation of disrupted neurobiological pathways, cooccurring with neuropsychiatric and other clinical symptoms.

Finally, our analysis also demonstrated that lower resilience was associated with poorer quality of life of the Lewy body disease group. This is consistent with evidence derived from older adults without neurodegenerative disorders, which suggests that the negative impact of stress and illness on quality of life can be mitigated by higher levels of resilience [60]. We have previously shown that the emergence of dementia in the context of Parkinson’s is associated with a significant increase in functional dependence of the person with Parkinson’s and lowered quality of life and higher care burden in care partners [3,61]. An important strength of our study was the focus on people with Lewy body-related cognitive disorders and their care partners, extending previous studies which examined people with Parkinson’s disease alone, without consideration of their cognitive stage (i.e., [16]).

We acknowledge certain limitations of our study. The lack of a universally accepted definition of resilience as a concept is already well recognised; however, some investigators contend that it is unnecessary to seek a single, unitary definition of resilience (i.e., [29]) and that contextual definitions suffice, provided the context is explicitly stated [62]. Moreover, assessing resilience using a unidimensional measure may be reductionist, considering the multidimensional nature of the concept. Despite this, our measure, the BRS, has previously demonstrated good psychometric and clinimetric properties [41]. Finally, the sample size of our study was relatively small, and only limited conclusions could be drawn from the study (including lack of comparisons between disease stages); however, we explored resilience in a complex neurodegenerative disorder and described care partners’ resilience in detail for the first time.

In the future, research should focus on enhancing understanding of the role of resilience with this population and examine in greater depth potentially relevant predisposing (e.g., personal traits) or enabling (e.g., self-care, services, and social support) factors that may influence resilience. Additionally, understanding resilience across different stages of the condition and in different care settings will also be important and informative for clinical care.

## 5. Conclusions

Our exploratory study is unique in that it has examined the role of resilience in the management of persons with Parkinson’s disease and dementia with Lewy bodies. This study also described the associations between resilience and mental health, neuropsychiatric symptoms, and quality of life in people with Lewy body-related cognitive disorders and their care partners. Assessing the level of resilience and focussing interventions on enhancing the skills to bounce back from stressful situations could help with supporting psychological well-being and reduce carer burden. The inclusion of mild cognitive impairment in Parkinson’s in our study is important in the development of an appropriate care plan. Future studies should include a larger sample size with a qualitative component to fully explore the concept of resilience among dyads affected by Lewy body spectrum disorders.

## Figures and Tables

**Table 1:** Descriptions and values of measures in people with Lewy body-related cognitive disorders and care partners (*n* = 76 participant-dyads).

Measures	Scale Description					Results		
						Mean (SD), Range		Mann–Whitney U Test
	Aims of the Measure	Completed by Whom	Number of Items	Scoring	Max Score	People with Lewy Body Cognitive Disorders	Care Partners	
Brief Resilience Scale (BRS)	Capacity to bounce back from stress	Both participants	6	5-point Likert, 1–5 ^a^	5.00	3.23 (0.71), 1.33–4.67	3.79 (0.82), 1.50–5.00	0.000 ***
Relationship Satisfaction Scale (RSS)	Communication, conflict resolution, degree of affection, intimacy, and overall relationship satisfaction	Both participants	7	7-point Likert, 0–6 ^a^	42	33.16 (7.47), 12–42	29.05 (10.36), 2–42	0.016 *
Hospital Anxiety and Depression Scale (HADS)	Anxiety, depression	Both participants	7 anxiety	4-point Likert, 0–3 ^b^	21	7.26 (4.15), 0–19	5.77 (4.31), 0–18	0.021 *
			7 depres- sion	4-point Likert, 0–3 ^b^	21	6.38 (2.73), 0–13	4.34 (3.87), 0–17	0.000 ***
EuroQoL-5D-3L (EQ5D)	Health-related quality of life	Both participants	5 (index score)	3-point Likert, 1–3 ^a^	1.000	0.541 (0.32), −0.113–1.000	0.806 (0.22), −0.016–1.000	0.000 ***
			1 (visual analogue scale)	0–100% ^a^	100%	64.93 (16.56), 25–95	75.90 (15.45), 35–100	0.000 ***
Parkinson’s Disease Questionnaire-39 (PDQ-39)	PD-specific health-related quality of life in 8 dimensions	Interview with people with PD	39	0–100% ^b^	100%	34.43 (14.69), 6.93–77.50	NA	NA
Neuropsychiatric Inventory (NPI)	Frequency and severity of 12 neuropsychiatric symptoms	Proxy rated by care partners	12	Frequency (1–4) × severity (1–3) ^b^	144	16.17 (14.34), 0–58	NA	NA
Zarit Burden Interview (ZBI)	Degree of burden	Care partners only	22	5-point Likert, 0–4 ^b^	88	NA	31.64 (16.06), 2–74	NA
Relatives’ Stress Scale (Rel.SS)	Amount of upset and stress experienced by the care partner due to care provision	Care partners only	15	5-point Likert, 0–4 ^b^	60	NA	22.21 (11.22), 0–55	NA
Short-Form 12 Health Survey (SF-12)	Physical and mental health	Care partners only	6 physical health	Binary (yes/no) or Likert ^a^	100	NA	49.80 (10.18), 24.34–66.80	NA
			6 mental health	Binary (yes/no) or Likert ^a^	100	NA	47.75 (11.48), 17.01–62.85	NA

^a^—higher scores better, ^b^—lower scores better, NA—not applicable, PD—Parkinson’s disease, SD—standard deviation. Notes: *** *p* < 0.001, * *p* < 0.05.

**Table 2:** Demographic and clinical variables of people with Lewy body-related cognitive disorders and care partners (*n* = 76 dyads).

		People with Lewy Body Cognitive Disorders (*n* = 76)		Care Partners (*n* = 76)	
**Categorical Variables**		***n***	**%**	***n***	**%**
Gender	Female	16	21.1	68	89.5
	Male	60	78.9	8	10.5
Ethnicity	White	71	93.4	70	92.1
	Nonwhite	4	5.3	5	6.6
	Did not specify	1	1.3	1	1.3
Education level	Up to 18 year old schooling	40	52.7	37	48.6
	Further education and higher	36	47.3	39	51.4
Marital status	Single	12	15.8	13	17.2
	Married/ Partnership	64	84.2	63	82.8
Living status	With others	70	92.1	74	97.4
	Alone	6	7.9	2	2.6
Diagnosis	PD-MCI	15	19.8		
	PDD	40	52.6		
	DLB	21	27.6		
Dyad relationship	Spouse/partner			59	77.6
	Son/daughter			13	17.1
	Other			4	5.3
Caregiving weekly hours (up to an average of)	1 h per day			15	19.7
	8 h per day			22	28.9
	24 h a day			39	51.4
H&Y stage	I	15	19.7		
	II	33	43.4		
	III	12	15.8		
	IV	12	15.8		
	V	4	5.3		
**Continuous Variables**		***n***	**Median; IQR (Range)**	***n***	**Median; IQR (Range)**
Age, years		76	75; 71–78 (55–90)	76	68; 59–72 (21–88)
Dyad known years (if spouses/partners)				63	48; 38–55 (0.50–70)
Duration of clinical symptoms, years		76	4.5; 2–10 (0–33)		
Caregiving years				76	3; 1–6.75 (0–20)
Montreal Cognitive Assessment (MoCA)		71	19; 15–22 (7–30)		
UPDRS-III		75	31; 18–40 (8–69)		
SE-ADL		74	60; 30–80 (10–100)		

DLB—dementia with Lewy bodies, H&Y—Hoehn and Yahr Staging, IQR—interquartile range, PD-MCI—Parkinson’s disease mild cognitive impairment, PDD—Parkinson’s disease dementia, SE-ADL—Schwab and England Activities of Daily Living, UPDRS-III—Unified Parkinson’s Disease Rating Scale-III.

**Table 3:** Outcome scores stratified by low and high resilience groups in people with Lewy body-related cognitive disorders and care partners (*n* = 76 dyads).

	People with Lewy Body-Related Cognitive Disorders				Care Partners			
Scales (Mean, SD)	Overall (*n* = 76)	Low (1.00–2.99) (*n* = 20)	High (3.00–5.00) (*n* = 56)	Mann–Whitney U Test *p*	Overall (*n* = 76)	Low (1.00–2.99) (*n* = 13)	High (3.00–5.00) (*n* = 63)	Mann–Whitney U Test *p*
RSS	33.16 (7.47)	32.10 (7.56)	33.54 (7.47)	0.350	29.05 (10.36)	21.00 (9.35)	30.71 (9.82)	**0.002**
HADS-Anxiety	7.26 (4.15)	10.65 (4.10)	6.05 (3.46)	**0.000**	5.77 (4.31)	11.69 (3.47)	4.54 (3.36)	**0.000**
HADS-Depression	6.38 (2.73)	7.30 (2.70)	6.05 (2.69)	0.104	4.34 (3.87)	8.46 (4.26)	3.49 (3.21)	**0.000**
EQ5D-index	0.54 (0.32)	0.47 (0.25)	0.57 (0.31)	0.318	0.81 (0.22)	0.60 (.30)	0.85 (0.18)	**0.001**
EQ5D-VAS	64.93 (16.56)	56.85 (14.01)	67.82 (16.55)	**0.004**	75.90 (15.45)	66.06 (10.73)	77.93 (15.56)	**0.003**
PDQ-39	34.43 (14.69)	43.23 (17.60)	31.29 (12.20)	**0.006**	NA	NA	NA	NA
NPI	16.17 (14.34)	22.40 (16.48)	13.95 (12.94)	**0.047**	NA	NA	NA	NA
ZBI	NA	NA	NA	NA	31.64 (16.06)	49.23 (15.90)	28.01 (13.59)	**0.000**
Rel.SS	NA	NA	NA	NA	22.21 (11.21)	34.85 (11.89)	19.60 (9.19)	**0.000**
SF-12-PCS	NA	NA	NA	NA	49.80 (10.18)	44.20 (12.58)	50.96 (9.32)	**0.037**
SF-12-MCS	NA	NA	NA	NA	47.75 (11.48)	34.81 (9.25)	50.42 (10.02)	**0.000**

BRS—Brief Resilience Scale; EQ-5D—EuroQoL-5D index score or visual analogue scale (VAS); HADS—Hospital Anxiety and Depression Scale, anxiety or depression subscale; NPI—Neuropsychiatric Inventory; PDQ-39—Parkinson’s Disease Questionnaire; Rel.SS—Relatives’ Stress Scale; RSS—Relationship Satisfaction Scale; SF-12—Short-Form 12 Health Survey, physical health (PCS) or mental health (MCS) subscale; ZBI—Zarit Burden Interview.

**Table 4:** Spearman correlation analyses among characteristics of people with Lewy body-related cognitive disorder and clinical outcomes (*n* = 76).

	BRS	Age	Clinical Symptoms (ys)	H&Y	UPDRS-III	SE-ADL	MoCA	RSS	HADS-A	HADS-D	EQ5D-index	EQ5D-VAS	PDQ-39
Age	0.132												
Duration of clinical symptoms	0.116	−0.163											
H&Y	−0.081	−0.027	0.387 **										
UPDRS- III	−0.295	0.000	0.351 **	0.539 ***									
SE-ADL	0.127	0.120	−0.303	−0.652 ***	−0.609 ***								
MoCA	0.051	−0.160	0.142	−0.185	−0.221	0.371 **							
RSS	0.267	0.352 **	−0.156	−0.187	−0.150	0.198	0.060						
HADS-A	−0.522 ***	0.276	0.131	0.134	0.050	−0.070	0.055	−0.400 ***					
HADS-D	−0.223	−0.340 **	0.264	0.320	0.218	−0.354 **	−0.019	−0.383 **	0.497 ***				
EQ5D- index	0.105	0.173	−0.293	−0.430 ***	−0.465 ***	0.566 ***	−0.072	0.119	−0.205	−0.437 ***			
EQ5D- VAS	0.399 ***	0.331**	−0.145	−0.184	−0.243	0.242	−0.005	0.315	−0.417 ***	−0.400 ***	0.388 **		
PDQ-39	−0.362 **	−0.252	0.356 **	0.402 ***	0.471 ***	−0.418 ***	0.002	−0.285	0.360 **	0.420 ***	−0.506 ***	−0.389 **	
NPI-total	−0.163	−0.174	0.199	0.179	0.284	−0.340 **	−0.073	−0.162	0.267	0.313	−0.366 **	−0.142	0.463 ***

** *p* < 0.002, *** *p* < 0.001 (Bonferroni adjustment applied). Abbreviations: BRS—Brief Resilience Scale; EQ-5D—EuroQoL-5D index score or visual analogue scale (VAS); H&Y—Hoehn and Yahr scale; HADS—Hospital Anxiety and Depression Scale, anxiety or depression subscale; MoCA—Montreal Cognitive Assessment; NPI—Neuropsychiatric Inventory; PDQ-39—Parkinson’s Disease Questionnaire; RSS—Relationship Satisfaction Scale; SE-ADL—Schwab and England Activities of Daily Living scale; UPDRS-III—Unified Parkinson’s Disease Rating Scale part III.

**Table 5:** Spearman correlation analyses among care partners’ characteristics (*n* = 76).

	BRS	Age	Cg hrs	Cg yrs	RSS	ZBI	HADS-A	HADS-D	SF12 PCS	SF12 MCS	EQ5D index	EQ5D VAS
Age	0.036											
Caregiving weekly hours	−0.108	−0.036										
Caregiving years	−0.213	0.183	0.310									
RSS	0.350 **	0.143	−0.200	−0.023								
ZBI	−0.444 ***	−0.319	0.290	0.191	−0.608 ***							
HADS-A	−0.654 ***	−0.043	0.036	0.194	−0.434 ***	0.624 ***						
HADS-D	−0.543 ***	−0.042	0.139	0.192	−0.535 ***	0.549 ***	0.696 ***					
SF-12-PCS	0.178	−0.096	−0.081	−0.079	0.114	−0.012	−0.199	−0.290				
SF-12-MCS	0.557 ***	0.172	−0.252	−0.127	0.486 ***	−0.553 ***	−0.709 ***	−0.654 ***	0.014			
EQ5D-index	0.348 **	−0.111	−0.093	−0.169	0.32	−0.226	−0.510 ***	−0.536 ***	0.651 ***	0.399 ***		
EQ5D-VAS	0.382 **	−0.094	−0.187	−0.101	0.265	−0.187	−0.377 **	−0.346 **	0.604 ***	0.379 **	0.518 ***	
Rel.SS	−0.446 ***	−0.253	0.423 ***	0.242	−0.622 ***	0.865 ***	0.623 ***	0.604 ***	−0.028	−0.669 ***	−0.296 ***	−0.218

** *p* < 0.003, *** *p* < 0.001 (Bonferroni adjustment applied). Abbreviations: BRS—Brief Resilience Scale; Cg—caregiving hours or years; EQ-5D—EuroQoL-5D index score or visual analogue scale (VAS); HADS—Hospital Anxiety and Depression Scale, anxiety or depression subscale; NPI—Neuropsychiatric Inventory; Rel.SS—Relatives’ Stress Scale; RSS—Relationship Satisfaction Scale; SF-12—Short-Form 12 Health Survey, physical health (PCS) or mental health (MCS) subscale; ZBI—Zarit Burden Interview.

**Table 6:** Linear regression analyses of resilience and key outcomes in people with Lewy body-related cognitive disorders and care partners and care partners.

	Resilience in People with People with Lewy Body-Related Cognitive Disorders							Resilience in Care Partners						
	*B*	*SE B*	β	*t*	95% CI	Adjusted R^2^	F-Value	*B*	*SE B*	β	*t*	95% CI	Adjusted R^2^	F-Value
EQ-5D-index	0.04	0.05	0.08	0.68	−0.069… 1.40	−0.007	0.467	0.10	0.03	0.37	3.38 **	0.041 … 0.158	**0.122**	11.434 **
EQ-5D-VAS	7.47	2.56	0.32	2.92	2.368 … 12.573	**0.091**	8.511 **	6.00	2.07	0.32	2.91 **	1.883 … 10.111	**0.090**	8.437 **
HADS-A	−2.68	0.60	−0.46	−4.47 ***	−3.881 … −1.487	**0.202**	19.971 ***	−3.58	0.44	−0.68	−8.05 ***	−4.461 … −2.691	**0.460**	64.859 ***
HADS-D	−0.80	0.44	−0.21	−1.83	−1.666 … 0.069	0.031	3.360	−2.57	0.46	−0.55	−5.64 ***	−3.482 … −1.665	**0.291**	31.849 ***
RSS	2.43	1.19	0.23	2.05 *	0.069 … 4.798	**0.041**	4.207 *	4.90	1.35	0.39	3.64 **	2.214 … 7.577	**0.140**	13.233 **
PDQ-39	−7.53	2.23	−0.37	−3.38	−11.981 … −3.086	**0.122**	11.392 **	N/A						
ZBI	N/A							−9.75	1.96	−0.50	−4.98	−13.661… −5.847	**0.240**	24.749 ***
Rel.SS								−7.07	1.35	−0.52	−5.22 ***	−9.761 … −4.371	**0.260**	27.290 ***
SF-12 PCS								3.26	1.39	0.26	2.35 *	0.496 … 6.017	**0.057**	5.526 *
SF-12-MCS								7.57	1.36	0.54	5.57 ***	4.859 … 10.275	**0.286**	31.009 ***

* *p* < 0.05, ** *p* < 0.01, *** *p* < 0.001. Abbreviations: B—unstandardised beta; β—standardised beta; SE B—standard error of unstandardised beta; t—*t*-test statistic; EQ-5D, EuroQoL-5D index score or visual analogue scale (VAS); HADS—Hospital Anxiety and Depression Scale, anxiety or depression subscale; NPI—Neuropsychiatric Inventory; Rel.SS—Relatives’ Stress Scale; RSS—Relationship Satisfaction Scale; SF-12 MCS—Short-Form 12 Health Survey, mental health subscale.

## Data Availability

The data presented in this study are available on reasonable request from the corresponding author. The data are not publicly available due to ethics regulations.
